# Profiling and Molecular Identification of Fungi Isolated From Maize Cultivated in Different Agroecological Zones in Nigeria

**DOI:** 10.1155/ijfo/9993093

**Published:** 2025-11-11

**Authors:** Edzili Awono Antoine Thierry, Ifeanyi Famous Ossamulu, Hadiza Kudu Muhammad, Isa Abdullahi Bala, Auta Helen Shnada, Susan Bekosai Salubuyi, Dogo Eustace, Shingu Jesse Polly, Hadiza Lami Muhammad, Essia Ngang Jean Justin, Makun Hussaini Anthony

**Affiliations:** ^1^ Africa Centre of Excellence for Mycotoxins and Food Safety, Federal University of Technology, Minna, Nigeria, futa.edu.ng; ^2^ Department of Microbiology, Faculty of Science, University of Yaoundé I, Yaoundé, Cameroon, uy1.uninet.cm; ^3^ Department of Biochemistry, Federal University of Technology, Minna, Nigeria, futa.edu.ng; ^4^ Department of Chemistry, Federal University of Technology, Minna, Nigeria, futa.edu.ng; ^5^ Department of Microbiology, Federal University of Technology, Minna, Nigeria, futa.edu.ng; ^6^ Department of Computer Engineering, Federal University of Technology, Minna, Nigeria, futa.edu.ng

**Keywords:** agroecological zone, fungi, maize, molecular characterization

## Abstract

This study investigated the fungal distribution on maize across Nigeria′s diverse agroecological zones. A total of 270 maize samples were collected from farms (90), markets (90), and storage facilities (90) from all seven agroecological zones in the country. The fungal strains were identified at the species level using conventional identification techniques, molecular methods, and phylogenetic analysis. The results showed that the highest fungal loads were recorded in the Sahel savanna (SHS), Sudan savanna (SS), and northern Guinea savanna (NGS) zones, with NGS showing peaks above 4.0 × 10^6^ CFU/g, particularly in farm and store samples. Lower fungal loads were observed in the mid altitude, derived savanna (DS), and humid forest (HF) zones, with median values mostly below 5.0 × 10^5^ CFU/g. Notably, the variability and presence of outliers were more pronounced in the SHS, SS, and NGS zones, indicating inconsistent contamination levels. A total of 986 fungal isolates were obtained from across the different agroecological zones. The fungi strains were grouped into 10 fungal genera, namely, *Aspergillus* sp. (42. 87%), *Fusarium* sp. (33.50%), *Penicillium* sp. (18.32%), *Rhizopus* sp. (3.46%), *Absidia* sp. (0.5%), *Mucor* sp. (0.5%), *Curvularia* sp. (0.3%), *Microsporum* sp. (0.1%), *Alternaria* sp. (0.1%), and *Cladosporium* sp. (0.1%). The molecular‐based identification of some of the isolates revealed the presence of new species in the crop, *Talaromyces sayulitensis*, *Aspergillus montevidensis*, *Epicoccum sorghinum*, *Aspergillus piperis*, *Exserohilum rostratum*, and *Tyrophagus putrescentiae*. The studies demonstrated a high prevalence of mycotoxin‐producing fungi, particularly from the genera *Aspergillus*, *Fusarium*, and *Penicillium*, which pose serious health risks due to their potential to contaminate food supplies with harmful toxins like aflatoxins and fumonisins.

## 1. Introduction

Maize is Nigeria′s most extensively cultivated cereal crop, playing a vital role in ensuring food security. It contributes approximately 20% of the population′s calorie intake and fulfills 16% of the country′s protein needs [1]. Smallholder farmers are the primary cultivators of this crop, which is grown on more than 30 million hectares throughout Sub‐Saharan Africa [2]. In 2020, Nigeria produced 10 million tons of maize, which served as food and livestock feed, particularly for poultry [3]. Nigeria′s maize sector is estimated to generate over $1 billion in potential export earnings with smallholder annual incomes around $410 per farm [4]. Compared to other leading maize producers in Africa, South Africa′s maize market is significantly larger, valued at approximately $4.1 billion, driven by robust domestic consumption and exports that earned $1 billion in 2023 [5]. Ethiopia produces about 9.6 million tonnes annually, with its maize market growing toward several hundred million dollars as productivity improves [6]. Tanzania′s maize production of around 6 million tonnes supports a market valued roughly between $400 and $600 million annually [7].

Pathogenic fungi are a major cause of maize grain deterioration and losses during storage, especially under favorable growth conditions. These fungi can account for approximately 50%–80% of the damage to stored maize [8]. Common fungal genera associated with stored grains include *Aspergillus*, *Penicillium*, *Fusarium*, and certain xerophytic species, many of which produce harmful mycotoxins that compromise the quality and safety of the grains [9]. Consequently, these fungal species contaminate maize with mycotoxins such as aflatoxins, fumonisins, trichothecenes (especially deoxynivalenol), and zearalenone, which are the most critical in maize safety and regulation [10]. According to the European Union Commission Regulation (EU) 2023/915, maximum allowable limits in unprocessed maize grains are 5 *μ*g/kg for aflatoxin B_1_, 10 *μ*g/kg for total aflatoxins, 4000 *μ*g/kg for fumonisins (B_1_ + B_2_), 1750 *μ*g/kg for deoxynivalenol, and 350 *μ*g/kg for zearalenone [11]. Aflatoxins are predominantly produced by fungal species such as *Aspergillus flavus* and *Aspergillus parasiticus* [12]. Fumonisins, on the other hand, are synthesized by *Fusarium verticillioides*, *Fusarium proliferatum*, and *Fusarium fujikuroi*. The production of zearalenone is attributed to *Fusarium graminearum*, *Fusarium culmorum*, *Fusarium cerealis*, and *Fusarium equiseti*, while deoxynivalenol is produced by *Fusarium graminearum* and *F. culmorum* [13]. Several factors promote fungal growth and mycotoxin production in maize, including temperatures between 20°C and 35°C, high relative humidity above 70%, and grain moisture content exceeding 13%–15%, extended storage under warm (25°C–35°C) and humid conditions [14]. The likelihood of mycotoxin contamination increases when inadequate farming techniques are employed, such as drying crops directly on the ground or using unsanitized tarpaulins. Poor storage sanitation, the absence of proper storage infrastructure, and a failure to adhere to sound agricultural practices also exacerbate the risk [15].

Consuming maize contaminated with mycotoxins poses significant health risks to both humans and animals, potentially triggering a range of severe toxic effects. Mycotoxin exposure can result in both acute and chronic health problems and, in some instances, may cause death. The main exposure routes to exposing the human body are by ingestion of contaminated food or feed, inhalation of spores, or dermal contact with contaminated materials [16, 17].

Nigeria′s diverse agroecological zones for maize cultivation include the Sudan savanna (SS) characterized by low rainfall (600–900 mm) and sandy–loam soils, the northern Guinea savanna (NGS) and southern Guinea savanna (SGS) zones with moderate to high rainfall (900–1500 mm) and loamy to sandy–clay soils, the derived savanna (DS) with moderate rainfall and variable soils, and the humid forest zone which experiences high rainfall (> 1500 mm) and fertile, well‐drained soils. Maize farming practices involve land selection favoring well‐drained sandy loam soils, timely land clearing with minimal topsoil disturbance, ridge planting with recommended spacings, and use of certified seeds. These variations in climate, soil types, and farming practices strongly influence fungal diversity, prevalence, and mycotoxin production in maize in Nigeria [18, 19].

The current study was aimed at determining the fungal profile of maize samples from stores, farms, and markets collected across the seven agroecological zones of Nigeria, considering the entire maize value chain. This comprehensive approach sought to identify the types and abundance of fungi contaminating Nigerian‐grown maize, providing critical insights into the public health risks associated with maize consumption in the country. This research provides novel insights into the current fungal diversity and contamination in maize within agroecological zones and value chain points increasingly affected by climate variability. By combining phenotypic and molecular methods along with phylogenetic analysis, this study delivers a comprehensive and current view of fungal populations impacting maize in Nigeria.

## 2. Methods

### 2.1. Study Location

**Figure 1:**
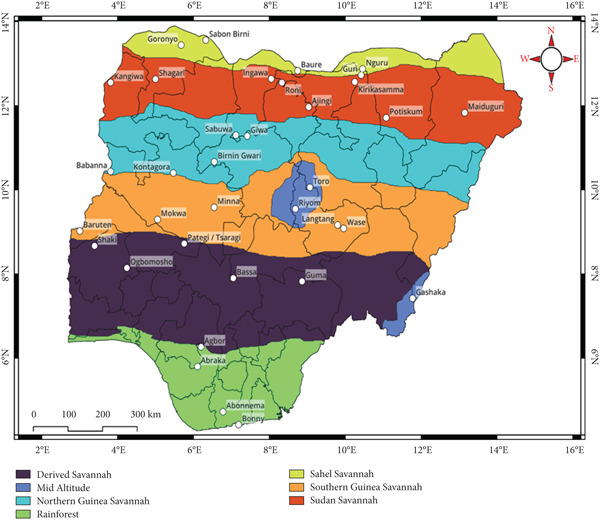
Overview of the agroecological zones with sampling sites.

The study location is Nigeria with sampling done within the seven agroecological zones: DS, SGS, NGS, mid altitude (MA), SS, Sahel savanna (SHS), and rain forest (Figure 1). The research was aimed at assessing the distribution of fungi in maize and was carried out at the African Centre of Excellence for Mycotoxin and Food Safety (ACEMFS), located at the Federal University of Technology, Minna, Nigeria.

### 2.2. Sample Collection

A total of 270 maize samples from Nigeria′s seven agroecological zones were collected during the dry season (February–April) 2024. In every district, we obtained samples from five selected locations of farms, in stores, and markets, each separated by an approximate distance of 20 km. From each site, we identified a farmer who had cultivated the crop in the preceding season and randomly sampled 1 kg of the crop, irrespective of the presence of visible fungal growth. After collection, samples were immediately transferred to sterile containers, stored in refrigerated cooler boxes, and transported to the ACEMFS. Table 1 shows the different locations and the weather conditions where the samples were collected, and Figure 1 shows the different agroecological zones of Nigeria with the sampling sites.

**Table 1:** Sampling sites in the seven agroecological zones (AEZs) of Nigeria [20].

**Zone**	**District (state)**	**Sample collected**	**Rainfall range (mm)**	**Average temperature range (°C)**
MA	Riyom, Lantang, Wase (Plateau state), Toro (Bauchi)	36	1300–1500	25–35
DS	Bassa (Kogi state), Gashaka (Taraba state), Guma (Benue state), Pategi/Tsaragi (Kwara state), and Ogbomosho	54	1000–1800	23–35
SGS	Shaki (Oyo state), Baruten (Kwara state), Mokwa (Niger state), Minna (Niger state), and Giwa (Kaduna state)	36	1000–1300	26–38
NGS	Sabuwa (Katsina state), Kontagora (Niger state), Birnin Gwari (Kaduna), Maiduguri (Borno state), and Babanna (Niger state)	36	900–1000	28–40
SS	Kangiwa (Kebbi state), Shagari (Sokoto state), Ingawa/Roni (Katsina/Jigawa), Ajingi (Kano state), and Potiskum (Yobe state)	36	650–1000	29–41
SHS	Goronyo, Sabon Birni (Sokoto state), Baure (Katsina), Kirikasamma (Yobe state), Guri/Nguru (Jigawa/Yobe)	36	450–1050	29–43
HF	Abraka, Agbor (Delta state) Abonnema, Bonny (River state)	36	1400–2700	22–30

Abbreviations: DS, derived savanna; MA, mid altitude; NGS, northern Guinea savanna; RF, rain forest; SGS, southern Guinea savanna; SHS, Sahel savanna; SS, Sudan savanna.

### 2.3. Sample Preparation

Before analysis, 250 g of each sample was milled using a sterile mechanical blender (Labinco, Breda, The Netherlands). To avoid any additional postharvest fungal growth, all the milled samples were stored in a freezer at −4°C.

### 2.4. Fungal Isolation and Macroscopic and Microscopic Identification

The fungal profiling of the collected maize samples followed the method described by Edzili et al. [21]. Briefly, 1 g of blended maize was suspended in 9 mL of sterile distilled water and vortexed vigorously for 2 min to homogenize the sample. Serial dilutions were prepared, and 100‐*μ*L aliquots from each dilution were spread onto antibiotic‐supplemented potato dextrose agar (PDA) plates. These were incubated at 28°C for 3–5 days, with colony‐forming units (CFUs) counted on Days 3–5. Microbial loads were calculated as CFU per gram of the sample, adjusting for dilution factors:

CFU/g=number of colonies×reciprocal of the dilution factorplating volumes mL

Distinct fungal colonies were then subcultured onto three different media: PDA, malt extract agar (MEA), and yeast extract agar (YEA) to obtain pure isolates. These isolates were stained with lactophenol cotton blue and examined microscopically using a compound microscope equipped with a digital camera (Unico, Model G508T, United States) for morphological identification. Identification was based on a combination of macroscopic colony characteristics and microscopic features according to established taxonomic keys for *Aspergillus* [22–24], *Penicillium*, and other genera [25].

### 2.5. Molecular Identification

Certain fungal isolates, which could not be identified taxonomically based on their phenotypic traits, were characterized through the sequencing of the Internal Transcribed Spacer (ITS) region of the nuclear ribosomal DNA (rDNA). This process utilized the universal primers ITS1 and ITS4 (as detailed in Table 2) to amplify the targeted ITS region [26].

**Table 2:** ITS primer and sequence.

**Name of primer**	**Target**	**Sequence (5** ^′^ **to 3** ^′^ **)**
ITS1	ITS rDNA sequence	TCCGTAGGTGAACCTGCGG
ITS4	ITS rDNA sequence	TCCTCCGCTTATTGATATGC

The genomic DNA from 10 selected fungal isolates was isolated using the Quick‐DNA Fungal/Bacterial Kit (Zymo Research, Cat. No. D6005). The ITS region was targeted for amplification with OneTaq Quick‐Load 2X Master Mix (NEB, Cat. No. M0486), employing primers detailed in Table 2. Following PCR, the amplicons were electrophoresed on an agarose gel and purified enzymatically through the EXOSAP protocol. Bidirectional sequencing was performed using the BrilliantDye Terminator Cycle Sequencing Kit V3.1 (NimaGen), followed by purification with the ZR‐96 DNA Sequencing Clean‐up Kit (Zymo Research, Cat. No. D4050).

Sequence analysis was conducted on an ABI 3500xl Genetic Analyzer (Applied Biosystems/Thermo Fisher Scientific), with raw data files processed through DNASTAR software. Consensus sequences were compared against the NCBI database using BLAST to confirm taxonomic identities. This workflow combines commercial kits for DNA isolation with enzymatic purification methods shown effective in reducing contaminants.

The phylogenetic analysis utilized a dataset comprising fungal isolates collected from agricultural crops and supplemented with published sequences sourced from GenBank. Consensus sequences were generated by aligning forward and reverse reads using Clustal W within the BioEdit software environment. Phylogenetic relationships were reconstructed employing the neighbor‐joining (NJ) algorithm, with evolutionary distances calculated via the maximum composite likelihood method. These distances are expressed as the number of nucleotide substitutions per site. All computational analyses were conducted using Molecular Evolutionary Genetics Analysis (MEGA) Software Version 11.0 [27].

## 3. Statistical Data Analysis

Data were analyzed using SPSS Statistics Version 23 to calculate the mean, standard deviation, and median values of the fungi load in each agroecological zone, while Microsoft Office Excel Professional Plus 2016 (Redmond, Washington, United States) was used for editing and formatting tables.

## 4. Results

### 4.1. Fungal Load

**Figure 2:**
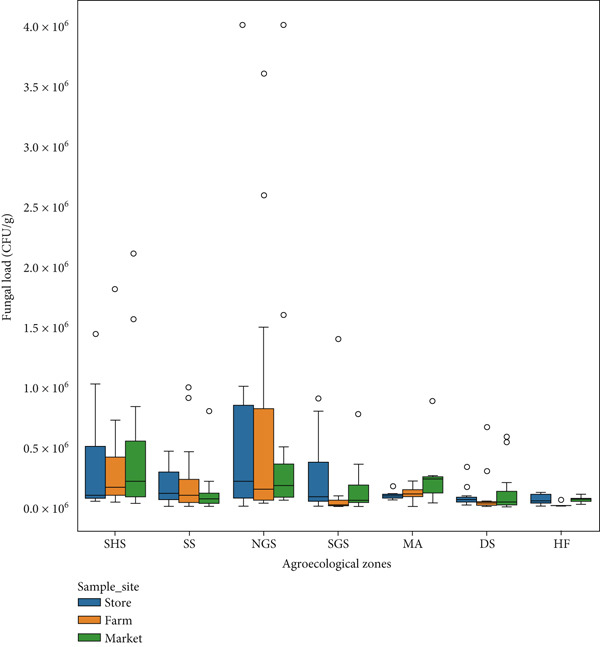
Fungi load in stores, farms, and market samples from different agroecological zones.

The boxplot graph (Figure 2) provides a comprehensive visualization of fungal load distribution, measured in CFUs per gram, across seven agroecological zones while comparing three critical value chain stages: farm, market, and store.

Among all zones, NGS exhibits the highest fungal contamination, with store samples reaching median values of approximately 1.1 × 10^6^ CFU/g with a mean of 1.4 ± 1.0 × 10^5^ CFU/g, farm samples around 9.6 × 10^5^ CFU/g (mean: 1.3 ± 1.1 × 10^5^ CFU/g), and market samples close to 8.7 × 10^5^ CFU/g. Outliers in NGS further escalate up to 4.0 × 10^6^ CFU/g (mean: 6.2 ± 5.0 × 10^4^ CFU/g), underscoring severe fungal proliferation likely driven by warm, humid climates and poor postharvest practices. A similar trend is observed in SHS, where store and market samples show median fungal loads of roughly 6.5 × 10^5^ CFU/g (mean: 2.0 ± 2.2 × 10^5^ CFU/g) and 5.9 × 10^5^ CFU/g (mean: 7.6 ± 6.0 × 10^4^ CFU/g), respectively, while farm samples are slightly lower at 4.1 × 10^5^ CFU/g (mean: 8.2 × ±8.8 × 10^4^ CFU/g). In SS, fungal load remains relatively high but less variable, with median values for store, farm, and market samples ranging from 2.5 × 10^5^ to 4.7 × 10^5^ CFU/g with means of 1.4 × ±1.0 × 10^5^, 1.3 ± 1.1 × 10^5^, and 6.2 ± 5.0 × 10^4^ CFU/g, respectively, and moderate outliers extending beyond 9.0 × 10^5^ CFU/g. In SGS, the fungal load is moderately high with store samples averaging 3.6 × 10^5^ CFU/g, while farm and market levels hover around 2.1 × 10^5^ and 2.8 × 10^5^ CFU/g, respectively. In MA, farm and market samples show median levels around 1.2 × 10^5^ and 1.5 × 10^5^ CFU/g with means 1.6 ± 1.9 × 10^4^ and 6.0 ± 2.8 × 10^4^ CFU/g, respectively, with store samples slightly higher at 2.3 × 10^5^ CFU/g. DS follows a similar pattern, with store samples near 2.1 × 10^5^ CFU/g, farm samples at 1.0 × 10^5^ CFU/g, and market samples at 1.6 × 10^5^ CFU/g with means of 7.8 ± 7.0 × 10^4^, 9.5 ± 1.5 × 10^4^, and 1.2 ± 1.7 × 10^5^ CFU/g, respectively. The humid forest zone is the least contaminated across all stages, with extremely low fungal load medians of 6.3 × 10^4^ CFU/g for stores, 5.7 × 10^4^ CFU/g for farms, and 5.0 × 10^4^ CFU/g for market samples. The means were 2.9 ± 3.0 × 10^4^, 1.6 ± 2.0 × 10^4^, and 6.0 ± 2.8 × 10^4^ CFU/g, respectively. The absence of notable outliers in HF reinforces its superior control over fungal growth, possibly due to favorable environmental conditions or better postharvest practices.

Based on the categorization of Gimeno, in which samples can be categorized as good, regular, and bad, all agroecological zones except the humid forest are classified as “bad” in terms of fungal contamination at every stage of the value chain. Even in HF, contamination remains at a “regular” level, indicating the need for better control measures.

### 4.2. Fungal Profiling

**Figure 3:**
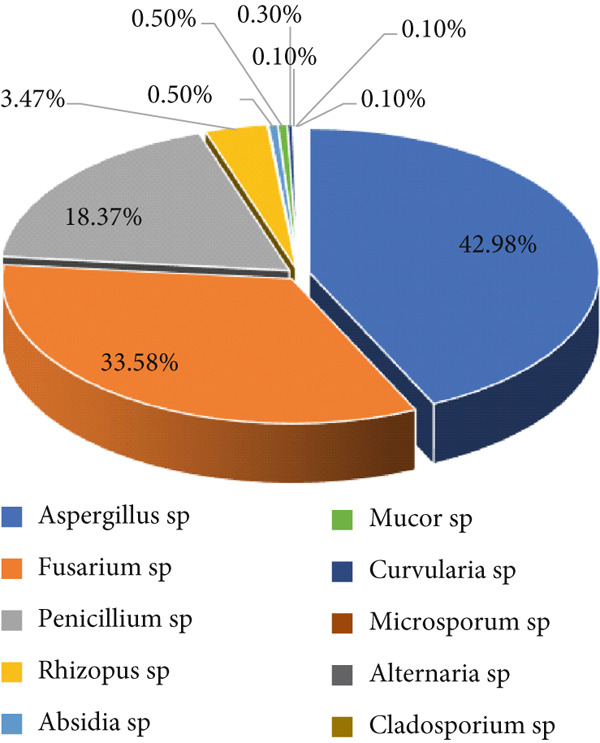
Fungi occurrence isolated in maize samples in Nigeria.

The findings on fungal contamination in maize samples gathered from storage facilities, farms, and markets across Nigeria′s seven agroecological zones are summarized in Table 3. Exactly 982 fungal species were isolated, all grouped into 10 different genera based on conventional identification. These were *Aspergillus* sp. (42.87%), *Fusarium* sp. (33.50%), *Penicillium* sp. (18.32%), *Rhizopus* sp. (3.46%), *Absidia* sp. (0.5%), *Mucor* sp. (0.5%), *Curvularia* sp. (0.3%), *Microsporum* sp. (0.1%), *Alternaria* sp. (0.1%), and *Cladosporium* sp. (0.1%). Figure 3 illustrates the frequency distribution of various fungal genera isolated across different agroecological zones in Nigeria.

**Table 3:** Fungi occurrence across the seven agroecological zones of Nigeria.

**Fungi isolates**	**SHS** **n** = 36	**SS** **n** = 36	**NGS** **n** = 36	**SGS** **n** = 36	**MA** **n** = 36	**DS** **n** = 54	**RF** **n** = 36	**Total** **n** = 270
*Aspergillus candidus*	1 (9.09)	1 (9.09)	1 (9.09)	3 (27.27)	2 (18.18)	3 (27.27)	0	11 (1.12)
*Aspergillus carbonarius*	0	1 (50.00)	0	0	0	1 (50.00)	0	2 (0.20)
*Aspergillus clavatus*	0	9 (64.28)	0	2 (14.28)	0	3 (21.42)	0	14 (1.42)
*Aspergillus flavus*	13 (9.77)	9 (6.76)	32 (24.06)	29 (21.08)	6 (4.51)	33 (24.81)	11 (8.27)	133 (13.54)
*Aspergillus fumigatus*	7 (25)	4 (14.28)	3 (10.71)	7 (25)	1 (3.57)	4 (14.28)	2 (7.14)	28 (2.85)
*Aspergillus nidulans*	1 (100)	0	0	0	0	0	0	1 (0.10)
*Aspergillus niger*	11 (10.47)	12 (11.42)	29 (27.61)	16 (15.23)	4 (3.80)	23 (21.90)	10 (9.52)	105 (10.69)
*Aspergillus ochraceus*	0	1 (8.33)	3 (25)	0	0	2 (16.66)	6 (50)	12 (1.22)
*Aspergillus oryzae*	1 (5.88)	2 (11.76)	0	4 (23.52)	2 (11.76)	3 (17.64)	5 (29.41)	17 (1.73)
*Aspergillus tamarii*	3 (6.81)	8 (18.18)	5 (11.36)	7 (15.90)	3 (6.81)	13 (29.54)	5 (11.36)	44 (4.48)
*Aspergillus terreus*	2 (25)	1 (12.5)	0	1 (12.5)	2 (40)	2 (40)	0	8 (0.81)
*Aspergillus parasiticus*	2 (4.76)	2 (4.76)	9 (21.42)	11 (26.19)	1 (2.38)	9 (21.42)	8 (19.04)	42 (4.27)
*Aspergillus versicolor*	1 (33.33)	0	0	2 (66.66)	0	0	0	3 (0.30)
*Aspergillus* spp.	0	0	0	1 (100)	0	0	0	1 (0.10)
*Penicillium citrinum*	9 (12.16)	5 (6.75)	19 (25.67)	15 (20.27)	4 (5.40)	16 (21.62)	6 (8.10)	74 (7.53)
*Penicillium chrysogenum*	2 (20)	0	1 (10)	3 (30)	2 (20)	2 (20)	0	10 (1.01)
*Penicillium commune*	0	0	0	0	3 (75)	1 (25)	0	4 (0.40)
*Penicillium griseofulvum*	1 (50)	0	0	1 (50)	0	0	0	2 (0.20)
*Penicillium notatum*	0	0	1 (100)	0	0	0	0	1 (0.10)
*Penicillium oxalicum*	0	4 (44.44)	0	3 (33.33)	0	2 (22.22)	0	9 (0.91)
*Penicillium pinophilum*	4 (12.90)	0	11 (35.48)	8 (25.80)	0	7 (22.58)	1 (3.22)	31 (3.15)
*Penicillium verrucosum*	7 (19.44)	2 (5.55)	5 (13.88)	9 (25)	7 (19.44)	6 (16.66)	0	36 (3.66)
*Penicillium* spp.	0	1 (7.69)	0	2 (15.38)	9 (69.23)	1 (7.69)	0	13 (1.32)
*Fusarium cerealis*	0	0	0	1 (20)	3 (60)	1 (20)	0	5 (0.50)
*Fusarium compactum*	0	0	0	0	1 (25)	0	3 (75)	4 (0.40)
*Fusarium equiseti*	1 (14.28)	1 (14.28)	1 (14.28)	3 (42.85)	1 (14.28)	0	0	7 (0.71)
*Fusarium graminearum*	2 (7.69)	1 (3.84)	7 (26.92)	8 (30.76)	1 (3.84)	4 (15.38)	3 (11.53)	26 (2.64)
*Fusarium nivale*	1 (100)	0	0	0	0	0	0	1 (0.10)
*Fusarium oxysporum*	22 (24.71)	12 (13.48)	22 (24.71)	14 (15.73)	4 (4.49)	12 (13.48)	3 (3.37)	89 (9.06)
*Fusarium proliferatum*	0	2 (9.52)	5 (23.80)	4 (19.04)	7 (33.33)	2 (9.52)	1 (4.76)	21 (2.13)
*Fusarium solani*	13 (13.40)	12 (12.37)	20 (20.61)	16 (16.49)	7 (7.21)	24 (24.74)	5 (5.15)	97 (9.87)
*Fusarium sporotrichioides*	1 (100)	0	0	0	0	0	0	1 (0.10)
*Fusarium verticillioides*	7 (9.33)	16 (21.33)	13 (17.33)	17 (22.66)	5 (6.66)	15 (20)	2 (2.66)	75 (7.63)
*Fusarium* spp.	0	0	1 (33.33)	0	0	2 (6.66)	0	3 (0.30)
*Absidia corymbifera*	0	0	1 (20)	0	4 (80)	0	0	5 (0.50)
*Alternaria* spp.	0	0	0	1 (100)	0	0	0	1 (0.10)
*Cladosporium oxysporum*	0	0	0	0	0	0	1 (100)	1 (0.10)
*Curvularia affinis*	0	0	0	1 (100)	0	0	0	1 (0.10)
*Curvularia clavate*	0	0	1 (50)	0	0	1 (50)	0	2 (0.20)
*Microsporum* spp.	0	0	0	1	0	0	0	1 (0.10)
*Mucor fragilis*	0	0	0	0	0	0	1	1 (0.10)
*Mucor* spp.	0	2 (50)	1 (25)	0	0	1 (25)	0	4 (0.40)
*Rhizopus arrhizus*	0	2 (66.66)	0	0	0	1 (33.33)	0	3 (0.30)
*Rhizopus sexualis*	0	0	0	0	0	0	2 (100)	2 (0.20)
*Rhizopus stolonifera*	0	0	0	1 (14.28)	0	1 (14.28)	5 (71.42)	7 (0.71)
*Rhizopus* spp.	3 (13.63)	6 (27.27)	2 (9.09)	1 (4.54)	9 (0.91)	1 (4.54)	0	22 (2.24)
Others	0	0	1 (50)	0	0	1 (50)	0	2 (0.20)
Total	115 (11.71)	116 (11.81)	194 (19.75)	192 (19.55)	88 (8.96)	197 (20.06)	80 (8.14)	982

*Note:* Values in brackets represent the percentage of incidence.

Abbreviations: DS, derived savanna; MA, mid altitude; *n*, number of samples collected; NGS, northern Guinea savanna; RF, rain forest; SGS, southern Guinea savanna; SHS, Sahel savanna; SS, Sudan savanna.

*Aspergillus* sp. was the most dominant genus among all isolated genera. Thirteen species were identified: *Aspergillus candidus*, *Aspergillus carbonarius*, *Aspergillus clavatus*, *A. flavus*, *Aspergillus fumigatus*, *Aspergillus nidulans*, *Aspergillus niger*, *Aspergillus ochraceus*, *Aspergillus oryzae*, *Aspergillus tamarii*, *Aspergillus terreus*, *A. parasiticus*, and *Aspergillus versicolor*. *A. flavus* was the most dominant species (13.54%) followed by *Aspergillus niger* (10.69%).

The second most dominant genus was *Fusarium* sp. with 10 species. These were *Fusarium cerealis*, *Fusarium compactum*, *Fusarium equiseti*, *Fusarium graminearum*, *Fusarium nivale*, *Fusarium oxysporum*, *Fusarium proliferatum*, *Fusarium solani*, *Fusarium sporotrichioides*, and *Fusarium verticillioides*. The most present species were *Fusarium solani* (9.87%), followed by *Fusarium oxysporum* (9.06%).

*Penicillium* sp. was the third most present genera. The height of its species was identified. These included *Penicillium citrinum*, *Penicillium chrysogenum*, *Penicillium commune*, *Penicillium griseofulvum*, *Penicillium notatum*, *Penicillium oxalicum*, *Penicillium pinophilum*, and *Penicillium verrucosum*. The most dominant species were *Penicillium citrinum* (7.53%), *Penicillium verrucosum* (3.66%), and *Penicillium pinophilum* (3.15%).

Regarding the agroecological zones, the most contaminated zones were DS (20.06%) followed by NGS (19.75%) and SGS (19.55%). *Fusarium oxysporum* was the most common species in SHS. *Fusarium verticillioides* was the most common species in SS. *A. flavus* was the most common species in NGS, SGS, DS, and RF. MA was dominated by *Penicillium* sp. (Table 2).

Moreover, *Penicillium notatum* was found to be particular in NGS, *Rhizopus sexualis* and *Cladosporium oxysporum* in RF, and *Curvularia affinis* and *Alternaria* sp. in SGS.

The isolated storage fungi (from market and store) were *Aspergillus* sp., *Fusarium* sp., *Penicillium* sp., *Rhizopus* sp., *Mucor* sp., *Absidia* sp., *Alternaria* sp., *Cladosporium* sp., and *Curvularia* sp. *Aspergillus oryzae* (52.94%) was the most prevalent species in store samples, *Penicillium pinophilum* (29.06%) was the most prevalent species in farm samples, and *A. parasiticus* (45.223%) was the most prevalent genera in market samples. Compared with the field fungi, we noticed that some genera were absent particularly *Alternaria* sp., *Cladosporium* sp., and *Curvularia* sp. in the field; these ones were found as particular at the storage level. We also observed the presence of *Microsporum* sp. to be particular as field genera and absent in the storage.

**Figure 4:**
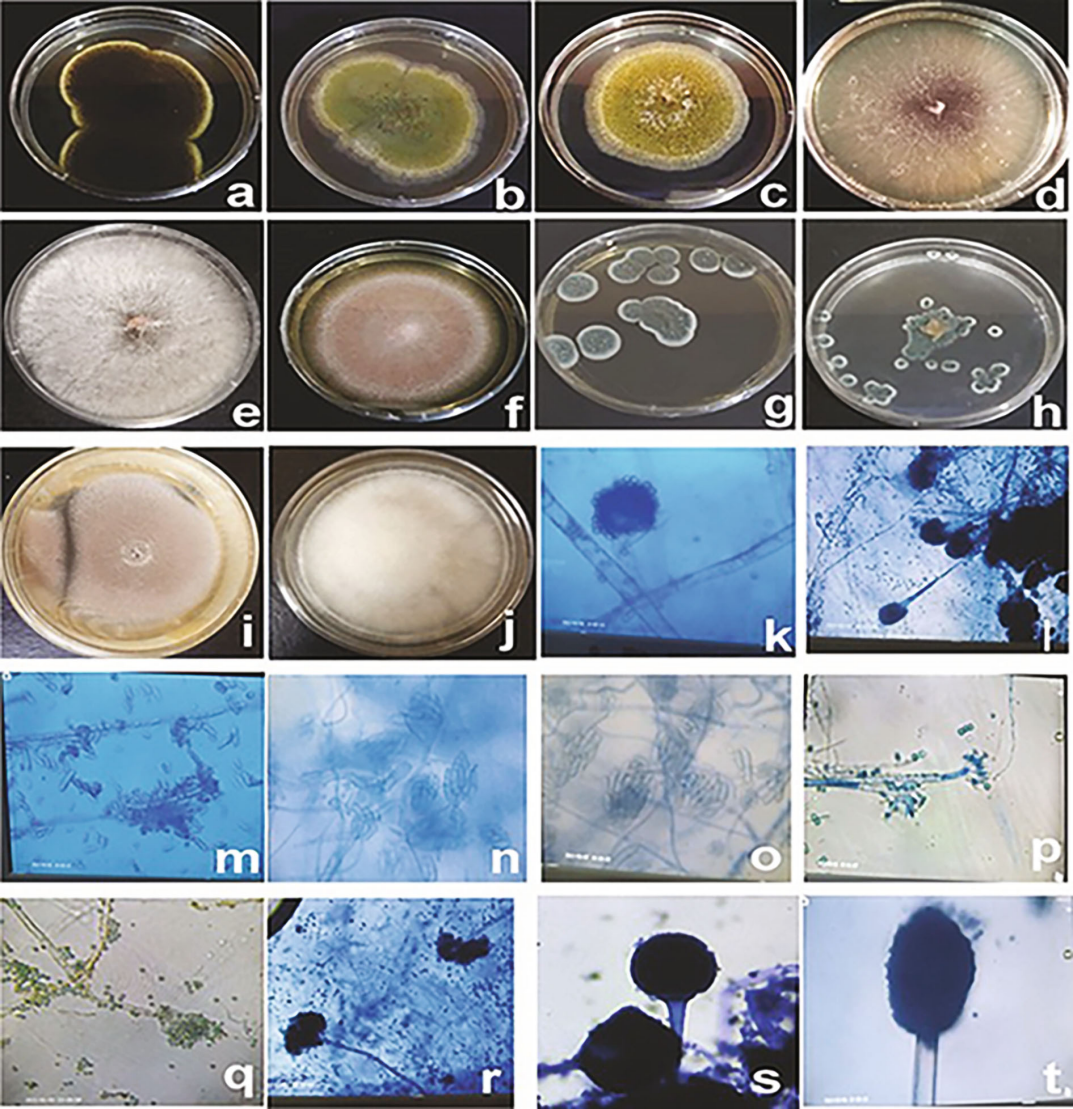
Figures of some fungi isolates from maize. (a, t) *Aspergillus niger*. (b, l) *Aspergillus flavus*. (c, k) *Aspergillus tamarii*. (d, m) *Fusarium oxysporum*. (e, n) *Fusarium solani*. (f, o) *Fusarium verticillioides*. (g, p) *Penicillium citrinum*. (h, q) *Penicillium verrucosum*. (i, r) *Aspergillus terreus*. (j, s) *Mucor* sp. (a–i) View of colonies after 7 days on PDA. (k–t) Observation of the fungi under the microscope.

**Figure 5:**
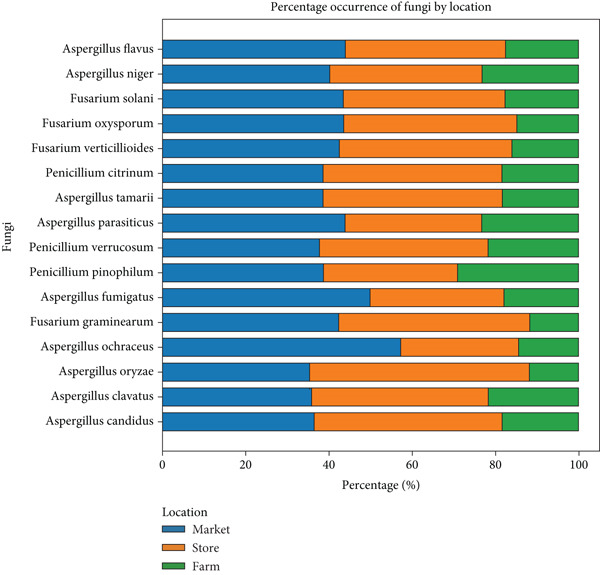
Distribution of fungal isolates from farm, store, and market across the agroecological zones.

Figure 4 illustrates both macroscopic and microscopic views of various fungal species isolated from the samples. Meanwhile, Figure 5 depicts the distribution of the most frequently occurring isolates across different agroecological zones in farms, stores, and markets. It indicates that most species such as *Aspergillus candidus*, *A. clavatus*, and *A. oryzae*, the store accounts for about 40% of the incidence, the farm for approximately 20%, and the market for about 40%. For isolates like *Aspergillus ochraceus*, *Fusarium graminearum*, *Penicillium pinophilum*, *P. verrucosum*, *P. citrinum*, *Fusarium verticillioides*, *F. oxysporum*, *F. solani*, *A. niger*, and *A. flavus*, the incidence is roughly 40% from the store, 30% from the farm, and 30% from the market. This pattern highlights that both store and market environments contribute equally and substantially to fungal contamination, while the farm generally shows a slightly lower incidence, suggesting that interventions to reduce fungal presence should focus on both the store and market environments, where the risk is highest.

### 4.3. Molecular Identification and Phylogenetic Analysis

A random selection of unidentified fungi using the conventional identification method of isolates revealed 10 genera, which later underwent molecular analysis. The fungal sequences that were amplified were utilized as queries for BLAST searches against the NCBI database. Table 4 presents the fungi closely related to those in GenBank, with most of them exhibiting a similarity range of 94%–100% to the corresponding fungi recorded in the database. These results revealed the presence of such species: *Talaromyces sayulitensis*, *Aspergillus niger*, *Aspergillus montevidensis*, *A. flavus*, *Epicoccum sorghinum*, *Aspergillus piperis*, *Curvularia lunata*, *Exserohilum rostratum*, and *Tyrophagus putrescentiae.*

**Table 4:** Characteristics of molecular‐based identified fungi.

**Sample ID**	**Origin**	**Organism**	**% of identity**	**Accession no. of BLAST hit**	**Highest query coverage (%)**
MF1	NGS (farm sample)	*Talaromyces sayulitensis*	98.81%	MZ014549.1	94%
RS2	SGS (store sample)	*Aspergillus niger*	99.47%	LT745387.1	100%
SF1	SS (farm sample)	*Aspergillus montevidensis*	100.00%	OP595962.1	99%
SS1	SHS (store sample)	*Aspergillus flavus*	99.33%	HQ340104.1	100%
RF1	DS (farm sample)	*Epicoccum sorghinum*	99.63%	ON204075.1	100%
RM1	RF (market sample)	*Aspergillus piperis*	99.34%	OL711715.1	100%
RM2	MA (market sample)	*Curvularia lunata*	100.00%	KY806118.1	100%
MM2	NGS (market sample)	*Exserohilum rostratum*	99.67%	ON074794.1	99%
MS1	SGS (store sample)	*Talaromyces sayulitensis*	100.00%	MZ014549.1	100%
RS1	DS (store sample)	*Tyrophagus putrescentiae*	79.82%	MF354009.1	99%

**Figure 6:**
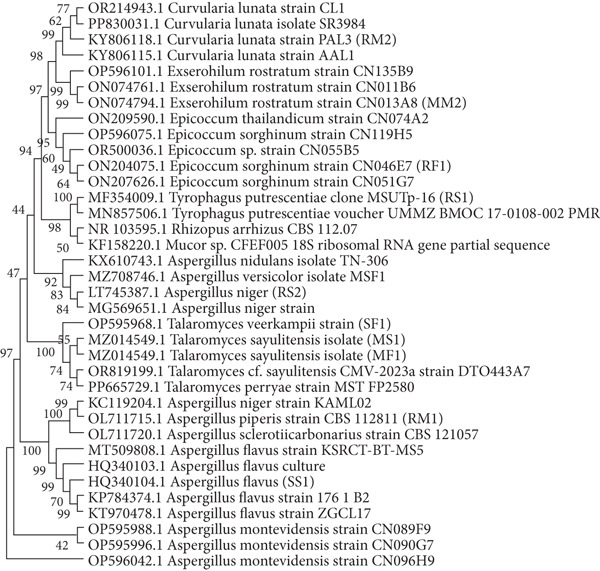
The evolutionary history of the molecular‐based identified fungi.

The phylogenetic analysis (Figure 6) revealed fungal isolates that could not be classified taxonomically using phenotypic traits but were identified through molecular methods. PCR‐amplified sequences exhibited 94%–100% similarity to 18S rRNA gene regions of closely related fungal genera and species in GenBank′s reference sequences.

## 5. Discussion

The high susceptibility of maize grain to fungal growth observed in this study had also been observed in different locations in Nigeria. Onyeche et al. [28] investigated the fungal and mycotoxin contamination of maize grains sourced from various senatorial zones in Benue state. Their findings revealed that a significant portion (40%) of maize samples from Zones A and B exhibited high levels of fungal contamination, with counts reaching or exceeding 3.1 × 10^7^ CFU/g. Akande et al. [29] analyzed the mycological and chemical screening of maize at open markets in Osun state. They found that the total fungi count ranged from 1.5 × 10^5^ to 2.1 × 10^7^ CFU/g in the six different studied locations. Gonzalez et al. [30] also investigated maize grain samples intended for animal feed and found total fungal counts ranging from 0 to 2.10 × 10^8^ CFU/g. The elevated level of contamination by various fungi can be ascribed to multiple factors, including the surrounding environmental conditions, moisture levels, and the inherent susceptibility of plants to fungal invasion. Furthermore, inadequate preharvest and postharvest practices, physical damage to grains caused by pests, improper drying techniques, and suboptimal grain storage conditions are potential contributing elements. Environmental variables, particularly elevated humidity and temperature, are recognized as facilitators of fungal proliferation in food and feed products [31]. Based on the meteorological data gathered from the sampled locations, most of the observations fall within the identified temperature and humidity ranges. The high levels of fungal contamination observed in this study could be explained by several factors. Unfavorable conditions during transportation and marketing may also contribute to the growth of fungi and the production of mycotoxins [32]. Mechanical damage sustained during or after harvesting may create pathways for fungal spore infiltration into maize cobs or grains, thereby increasing susceptibility to fungi infestation, as previously reported [33]. Insect damage, observed in numerous maize samples infested with weevils, likely exacerbates fungi growth. Insects attack maize both in the field and during storage, significantly contributing to fungi infection by either wounding the plant or serving as vectors for fungal spores [34].

An assessment of the contamination of the samples shows a diversity of fungi in dry maize as shown in Table 3. This fungal diversity is observed when moving from one agroecological zone to another and from one sampling site to another. This high diversity of different fungi species could be explained by the fact that cereals, and maize in particular, are preferred substrates for fungi because of their high carbohydrate content, recognized as the main source of energy [35]. This fungi contamination of samples could originate in the field or during storage [36]. In the fungi that develop, there are spores embedded in dry maize grains before storage. Drying before storage does not eliminate fungi spores but hinders their development. Furthermore, these fungi can develop in favorable temperature and humidity conditions [37]. Indeed, the climatic conditions (average temperature equal to 29°C) linked to these geographical locations as shown in Table 1 are favorable to the development of fungi in the field and during the storage of maize. The optimum temperature for fungi growth is between 20°C and 30°C [37].

All fungal species identified in this study are among the most common contaminants of maize in Nigeria. Like in Niger state [38], Anambra state [39], Kebbi state [40], Kebbi state [41], Ondo state [42], Lagos state [43], Ibadan [44], and some Nigerian agroecological zones (HF, NGS, SGS, and SS) [45].

*Aspergillus* sp. (42.87%) emerged as the predominant genus among all the isolated genera identified in this investigation. A total of 13 species were classified. The increased prevalence of *Aspergillus* observed in this study may be associated with the use of various maize storage methods. Examples of these methods include plastic or synthetic bags, piles on the floor, verandahs, fire racks, and outdoor structures such as granaries. Some of these storage techniques are inadequate in preventing moisture absorption and fungi growth, which compromises the protection of maize from aflatoxin contamination [46]. Higher moisture levels in some maize samples may have facilitated the growth of *Aspergillus* species. These fungi can thrive in grains with moisture as low as 15%, whereas moisture levels below 12%–13% generally inhibit fungal development regardless of temperature. This potentially indicates that *Aspergillus* species are afforded more conducive environmental conditions compared to the other fungal isolates in this study, as evidenced by their high frequency of occurrence. Significant concentrations of *Aspergillus* species have been reported in Nigeria, particularly in association with preharvest maize [47]. It is plausible that preharvest infections significantly impact the microflora present during storage [47].

In all seven agroecological zones, *Penicillium* and *Fusarium* species, alongside *Aspergillus*, were identified as fungi associated with maize. These genera are known to produce mycotoxins, and their presence has been documented extensively in maize cultivated in Nigeria [47], Benin [48], and Ghana [49]. The simultaneous presence of toxigenic fungi on commodities in stores, farms, and market places is frequently intensified as storage conditions become favorable for the synthesis of mycotoxins.

From the current study, the second most present genus was *Fusarium* sp. with 10 species. From the seven agroecological zones, the recurrent isolation of *Fusarium* species suggests a potential risk of maize contamination by fumonisins produced by *Fusarium oxysporum*. In certain regions, it has been documented that farmers frequently leave their maize harvests in the fields after maturation to facilitate the drying process. This practice may elucidate the prevalent incidence of *Fusarium* species detected within these maize specimens.

*Penicillium* species have a frequency of occurrence of 18.32% in all seven agroecological zones. The agroecological conditions in different locations can influence the prevalence of specific fungal genera over others. This aligns with previous research indicating that high soil temperatures and drought conditions are linked to higher rates of fungal infestation and the presence of mycotoxin‐producing species or strains [43].

The isolation of *Alternaria* species highlights its potential as a biological control agent against mycotoxigenic fungi, as evidenced by its ability to establish itself within the kernel ecological niche [50].

The market showed a higher occurrence of fungi, possibly due to the transportation and storage methods used. Grains are often moved to markets in locally crafted woven baskets and sacks, which can create conditions favorable for fungal growth. Additionally, the grains are frequently left uncovered in open containers at the market [51]. The variation in fungal incidence might be influenced by climate, and the types of fungi present could differ from those found in other regions.

At the farm level, a significant challenge is that maize contaminated by fungi often appears visually identical to healthy grains, showing no external signs of infection. Research has revealed that even seemingly good‐quality maize can be heavily infested with various fungi species. Unfortunately, farmers and crop handlers, particularly women, lack sufficient knowledge about proper harvesting, handling, and storage techniques. This deficiency leads to extensive damage caused by insect pests and fungal growth during storage and marketing. Additionally, considerable losses occur during crop processing. Reports indicate that in certain regions of Africa, cereal grains experience notable losses during harvesting, drying, and threshing stages [51]. In Zambia, maize dried on raised platforms experienced losses of 3.5%, while in Zimbabwe, the losses were slightly higher at 4.5%. For smallholder farmers in Zimbabwe using manual methods, threshing and shelling losses were estimated at 1%–2.5% and 3.5%, respectively, with mechanized shelling showing similar loss rates.

It was observed that DS (20.06%), NGS (19.75%), and SGS (19.55%) were the most contaminated agroecological zones (Table 3). The DS is characterized by a bimodal rainfall pattern range of 1000–18,000 mm (Table 1), which significantly affects soil moisture levels. The interplay of elevated temperatures and variable moisture creates an environment conducive to fungal growth. Rising temperatures in the region have further intensified the risk of fungal proliferation, as warmer climates typically enhance fungal activity and reproduction [52]. The main crops grown in the DS, such as maize, cassava, and yam, are highly vulnerable to numerous fungal diseases. The prevalence of these diseases is exacerbated by intensive farming methods, including monocropping and insufficient crop rotation, which allow pathogens to build up in the soil. This issue is further worsened by the limited use of fungicides or disease‐resistant crop varieties, largely due to economic challenges faced by local farmers [53].

A comparison of fungal isolates with sequences in the GenBank database revealed significant genetic similarity, indicating that closely related terrestrial fungal species are already well documented. This suggests limited evolutionary divergence among these species, which may have been sufficient to enhance their survival. Furthermore, the high genetic homology with previously identified isolates implies that these fungi have not encountered environmental or other pressures that would drive substantial genetic variation. This phenomenon aligns with the concept of concerted evolution [54]. The ability of closely related fungal species to colonize maize suggests a historical interaction with the crop, leading to the development of mechanisms enabling its degradation.

The genus *Talaromyces*, which currently includes over 170 recognized species, has been increasingly identified as an associate of insects in both agricultural and nonagricultural studies. To date, 27 species have been documented in association with insects from nine different orders, with this trend showing notable growth [55]. *T. sayulitensis*, identified in this study, has previously been isolated from the rhizosphere of pineapple, corn, and pepper in Indonesia. It has demonstrated the ability to infect and cause mortality rates ranging from 16.67% to 46.67% in cocoa bugs (*Helopeltis* sp.) [56]. *T. sayulitensis* is likely to produce mycotoxins similar to those found in *Talaromyces islandicus*, such as cyclochlorotine. Cyclochlorotine is synthesized via a nonribosomal peptide synthetase, indicating a complex biosynthetic pathway that may also exist in *T. sayulitensis* [57]. Cyclochlorotine is recognized for its hepatotoxic properties, leading to significant liver damage as evidenced by morphological changes in liver cells [58]. Studies indicate that cyclochlorotine induces dilatation of liver spaces and formation of membrane‐bound vacuoles, which are indicative of acute liver injury [58].

*A. montevidensis* is commonly isolated from various agricultural products, particularly nuts such as pistachios, walnuts, and hazelnuts, where it contributes to spoilage and potential mycotoxin contamination [59]. In Eastern Kenya, *A. montevidensis* was identified among other toxigenic species in maize and soil, highlighting its prevalence in agricultural settings [60]. In clinical settings, *A. montevidensis* has been implicated in infections such as otitis and pulmonary infections, indicating its potential pathogenicity alongside its mycotoxin production capabilities [61].

*A. piperis* can be isolated from maize kernels, where it is typically identified alongside other species such as *A. flavus* and *A. parasiticus*. Studies have shown that *Aspergillus* species are prevalent in maize, with *A. flavus* being the most common contaminant, followed by *A. parasiticus*. In some reports, *A. piperis* has been found in smaller quantities, indicating its presence within the diverse fungal community associated with maize [62, 63]. The black *Aspergilli* including *A. piperis* are recognized for their capacity to produce mycotoxins, which can present significant health hazards to both humans and animals. While *A. flavus* and *A. parasiticus* are more notorious for aflatoxin production, studies indicate that *A. piperis* may also contribute to mycotoxin contamination in agricultural products [64]. Interestingly, research has explored the antagonistic properties of *A. piperis* against certain phytopathogenic fungi. It has been found to produce lytic enzymes such as chitinase and protease, which may help it compete against other harmful fungi in agricultural settings [64]. This characteristic suggests a potential utility for *A. piperis* in biocontrol applications within crop management.

In 2020, *E. sorghinum* was identified as the cause of severe leaf sheath and leaf spot diseases in maize fields located in Jiangsu, China. The affected plants displayed red–brown lesions on their stems and leaves, resulting in significant damage. Approximately 95% of the maize plants in the observed area exhibited symptoms, leading to a dramatic reduction in crop yield, estimated at 70%–85% compared to previous years when no signs of the disease were present [65]. The pathogen′s ability to thrive in environments previously occupied by sorghum suggests a close ecological relationship between these crops, which may facilitate the spread of the fungus. Tenuazonic acid (TeA), produced by *E. sorghinum*, is a significant concern due to its toxicological implications. It can inhibit protein synthesis in plants, leading to growth disorders. In a study analyzing Argentinean sorghum samples, it was found that 65% of the isolates tested were capable of producing TeA at concentrations ranging from 112 to 47,237 *μ*g·kg^−1^ [66]. This contamination poses risks not only to plant health but also to livestock and potentially human health through contaminated food sources.

*Exserohilum rostratum* is a fungal pathogen recently identified as a causal agent of leaf spot disease in maize, particularly noted in Henan Province, China. This discovery marks a significant addition to the known pathogens affecting maize crops, highlighting the need for increased awareness and management strategies in agricultural practices. The disease manifests through distinct leaf spots that can coalesce into larger necrotic areas, severely affecting the plant′s health and yield [67, 68]. Further studies are necessary to explore the pathogen′s biology, virulence factors, and potential control measures [68]. In summary, *Exserohilum rostratum* poses a new threat to maize cultivation, necessitating proactive measures in agronomy and crop management to safeguard yields and maintain food security.

The findings of this study highlight the necessity of implementing effective fungi management strategies, including the application of fungicides and the use of physical and mechanical methods to create an environment unfavorable for fungal growth. Basic preventive measures, such as removing and destroying debris from previous harvests, are essential to reduce infection and infestation in maize. Sorting out physically damaged or infected seeds is also critical. Additionally, there is an urgent need to educate maize farmers, traders, and marketers in Nigeria about storage fungi and their management.

Improved storage facilities are vital for preserving maize grain quality. Adequate drying of maize to a moisture content below 13% is necessary to prevent biological activity, while eliminating insect activity is crucial to avoid moisture buildup caused by respiration, condensation, or low temperatures. The adoption of metal silo technology, as demonstrated by Gitonga et al. [69], has proven effective in controlling storage pests and fungi among small‐scale farmers. This technology not only reduces fungal contamination but also enhances food security in rural communities.

## 6. Conclusions

This study provided an extensive analysis of fungal distribution in maize across all agroecological zones of Nigeria. Using conventional identification techniques, molecular methods, and phylogenetic analysis, fungal species from 270 maize samples were identified. The research revealed the presence of common fungal species spanning 10 genera, distributed across seven agroecological zones. The molecular‐based identification revealed the presence of five new genera in the crop: *Talaromyces* sp., *Epicoccum* sp., *Exserohilum* sp., and *Tyrophagus* sp. *Aspergillus* sp., *Fusarium* sp., and *Penicillium* sp. were the most frequently isolated genera. Fungi were more frequently isolated in the dry zone compared to the temperate zones. The areas with the highest levels of contamination were DS, followed closely by NGS and then SGS. When we look at the origin of the samples, the ones from the market had a higher prevalence than stores and farms. Research has highlighted that even high‐quality maize can be significantly contaminated with various fungi species. This underscores the need to develop effective strategies and innovative solutions to enhance maize quality and safety, which in turn can boost the income and nutritional well‐being of farming households. The findings of this study are highly relevant to all stakeholders along the maize value chain, including farmers, middlemen, market vendors, storage facility operators, and policymakers. Understanding the fungal contamination patterns can guide targeted interventions at critical points especially storage and markets where contamination was highest. Adoption of good agricultural and postharvest practices such as thorough drying of maize before storage, use of clean and well‐ventilated storage facilities, regular monitoring for fungal growth, and implementation of Hazard Analysis and Critical Control Points (HACCP) can significantly reduce fungal proliferation and mycotoxin risk. Additionally, farmer education and government extension services play a vital role in raising awareness about proper maize handling techniques and the health risks associated with fungal contamination. Through such integrated efforts, the maize sector can improve grain safety and quality, minimize economic losses, and protect the health of consumers and farming communities alike. Nonetheless, this study has limitations. Sampling was confined to the dry season (February–April 2024), limiting understanding of seasonal variation in fungal contamination across zones. Moreover, the study did not examine the mycotoxin‐producing potential of the fungi isolated, nor distinguish between toxigenic and atoxigenic strains. These factors restrict conclusions about year‐round contamination risks and mycotoxin exposure. Future research should involve year‐round sampling to capture seasonal dynamics and employ molecular and biochemical assays to quantify mycotoxin production and detect toxigenic fungi. Investigations into the effectiveness of improved storage technologies and postharvest interventions under practical field conditions would provide actionable guidance for stakeholders. Such integrated research efforts will enhance the evidence base for policies and programs aimed at strengthening maize safety, food security, and public health in Nigeria.

## Disclosure

All authors read and approved the final manuscript.

## Conflicts of Interest

The authors declare no conflicts of interest.

## Author Contributions

E.A.A.T.: writing—original draft, methodology; I.F.O.: data analysis; H.K.M.: visualization, review and editing; I.A.B.: review and editing; A.H.S.: review and editing; S.B.S.: review and editing; D.E.: supervision and validation; S.J.P.: visualization; H.L.M.: supervision, resources; E.N.J.J.: visualization and supervision; M.H.A.: supervision, validation.

## Funding

The study is supported by the Tertiary Education Trust Fund, 10.13039/501100008895 (TETF/ES/DR&D‐CE/NRF2021/SETI/AFS/00335/01VOL.1).

## Data Availability

The datasets generated and/or analyzed during the current study are available from the corresponding author upon reasonable request.
